# Endothelial Injury Associated with Cold or Warm Blood Cardioplegia during Coronary Artery Bypass Graft Surgery

**DOI:** 10.1155/2015/256905

**Published:** 2015-05-19

**Authors:** Elmar W. Kuhn, Yeong-Hoon Choi, Jung-Min Pyun, Klaus Neef, Oliver J. Liakopoulos, Christof Stamm, Thorsten Wittwer, Thorsten Wahlers

**Affiliations:** ^1^Department of Cardiothoracic Surgery, Heart Center of the University of Cologne, Kerpener Strasse 62, 50924 Cologne, Germany; ^2^Center for Molecular Medicine Cologne, University of Cologne, Kerpener Strasse 62, 50924 Cologne, Germany; ^3^Department of Cardiothoracic Surgery, German Heart Institute Berlin, Augustenburger Platz 1, 13353 Berlin, Germany

## Abstract

The aim of this investigation was to analyze the impact of intermittent cold blood cardioplegia (ICC) and intermittent warm blood cardioplegia (IWC) on endothelial injury in patients referred to elective on-pump coronary artery bypass graft (CABG) surgery. Patients undergoing CABG procedures were randomized to either ICC or IWC. Myocardial injury was assessed by CK-MB and cardiac troponin T (cTnT). Endothelial injury was quantified by circulating endothelial cells (CECs), von Willebrand factor (vWF), and soluble thrombomodulin (sTM). Perioperative myocardial injury (PMI) and major adverse cardiac events (MACE) were recorded. Demographic data and preoperative risk profile of included patients (ICC: *n* = 32, IWC: *n* = 36) were comparable. No deaths, PMI, or MACE were observed. Levels of CK-MB and cTnT did not show intergroup differences. Concentrations of CECs peaked at 6 h postoperatively with significantly higher values for IWC-patients at 1 h (ICC: 10.1 ± 3.9/mL; IWC: 18.4 ± 4.1/mL; *P* = 0.012) and 6 h (ICC: 19.3 ± 6.2/mL; IWC: 29.2 ± 6.7/mL; *P* < 0.001). Concentrations of vWF (ICC: 178.4 ± 73.2 U/dL; IWC: 258.2 ± 89.7 U/dL; *P* < 0.001) and sTM (ICC: 3.2 ± 2.1 ng/mL; IWC: 5.2 ± 2.4 ng/mL; *P* = 0.011) were significantly elevated in IWC-group at 1 h postoperatively. This study shows that the use of IWC is associated with a higher extent of endothelial injury compared to ICC without differences in clinical endpoints.

## 1. Introduction

Cardiopulmonary bypass during cardiac surgical procedures is associated with myocardial and endothelial injury [1–3]. Myocardial protection in form of intermittent cold (ICC) and intermittent warm blood cardioplegic solutions (IWC) is still under investigation even though clinically introduced decades ago. Good clinical results were reported with ICC and IWC among other potential options for myocardial protection. The use of IWC was demonstrated to be favourable over ICC in low-risk patients referred to coronary artery bypass procedures, whereas ICC was shown to provide superior myocardial protection in high-risk populations requiring prolonged cross clamp times [4, 5].

Most trials investigating the impact of cardioplegic solutions failed to detect differences in patients' clinical outcome due to the limited direct impact of cardioplegia on hard clinical endpoints. However, the influence of myocardial protection techniques is well investigated on inflammatory processes [3]. Since inflammation correlates with the degree of endothelial injury we sought to investigate in the presented study endothelial function in patients undergoing coronary artery bypass graft (CABG) procedures using IWC or ICC. For the assessment of endothelial injury circulating endothelial cells (CECs), von Willebrand factor (vWF), and soluble thrombomodulin (sTM) were chosen as well established indicators since respective concentrations reflect the degree of endothelial injury associated with inflammation (vWF and sTM) and vascular damage (CECs) especially in patients with heart failure, diabetes, and various types of vasculitis [6–9]. In patients with acute myocardial infarction, recent data indicates that CEC counts may predict rupture of atherosclerotic plaque [10]. However, CEC count has never been established for evaluation of IWC and ICC in coronary artery bypass graft (CABG) patients.

We therefore aimed in the present study to test the hypothesis if the use of ICC is associated with a reduced endothelial injury in routine CABG procedures what might in part explain the favourable results associated with IWC over ICC.

## 2. Materials and Methods

### 2.1. Study Design and Patients

A randomized and blinded single-center pilot trial was performed of patients (*n* = 72) scheduled for elective coronary artery bypass graft (CABG) surgery using cardiopulmonary bypass (CPB) at the Heart Center of the University of Cologne between July 2013 and December 2013. Exclusion criteria were ejection fraction <25%, age >80 years, acute myocardial infarction, atrial fibrillation, combined or redo procedures, history of diabetes mellitus, renal or hepatic dysfunction, vasculitis, cancer, or infectious diseases as well as intake of drugs that are known to directly interact with the endothelium (e.g., antioxidants). Eligible patients were randomized into two groups: (1) intermittent cold blood cardioplegia- (ICC-) group and (2) intermittent warm blood cardioplegia- (IWC-) group using a 1 : 1 web-based and open-access randomization plan (generated by http://www.randomization.com/). Sample acquisition was incomplete in 4 patients in the ICC-group; these patients were not included into analysis. All patients were blinded for group allocation and data analysis was carried out without knowledge of group allocation. Intraoperative blinding of surgeons and perfusionists was not carried out. The study was approved by the ethics committee of the University of Cologne (Cologne, Germany) and all participating patients gave written informed consent.

### 2.2. Surgical Technique and Cardioplegic Delivery

Induction of anesthesia was performed with 1 *μ*g/kg sufentanil, 0.2 mg/kg etomidate, 0.04 mg/kg midazolam, and 0.15 mg/kg cisatracurium and maintained with sufentanil (1 *μ*g/kg/mh) and midazolam (0.1 mg/kg/h). Additional sevofluran was administered until CPB.

Surgical technique and perioperative management were uniform for all patients. All operations were performed on CPB consisting of a centrifugal pump, a membrane oxygenator (Jostra Quadrox, Maquet Cardiopulmonary AG, Hirrlingen, Germany), and an in-line arterial filter. Mean arterial pressure was maintained between 50 and 70 mmHg and CPB flow was kept at 2.2–2.5 L/min per m^2^. Core temperatures for patients in the IWC-group were kept at 37°C during CPB and moderate hypothermia (32–34°C) was applied for ICC-patients. Cardioplegia was chosen according to randomization.

In the ICC group, induction of cardiac arrest was achieved giving cold (4–6°C), nonsubstrate enriched Buckberg solution (Köhler Chemie GmbH, Alsbach-Hähnlein, Germany) for 4-5 min with perfusion pressures of 60 mmHg for antegrade cardioplegia delivery [2, 11]. The induction solution was supplemented with potassium to deliver a final concentration of ~20 mmol/L. In the IWC-group, warm (37°C) oxygenated blood was infused into the aortic root. A syringe pump containing a potassium and magnesium mixture (30 mL of 2 mmol/mL KCL; 10 mL of 2 mmol/mL MgSO_4_) was connected to the cardioplegia circuit. Cardiac arrest was induced at a blood flow rate of 200–300 mL/min by continuous infusion of 150 mL/h of the syringe pump over a 2-3 min time period [12, 13]. For both IWC and ICC, maintenance of cardiac arrest was facilitated by antegrade reinfusions into the aortic root for 3-4 min every 15–20 min or after completion of an anastomosis (cardioplegic delivery via completed bypass grafts is not routinely performed at our institution). Independently of applied cardioplegic strategy, warm reperfusion was always obtained by release of the aortic clamp. Proximal anastomoses were completed on a beating heart using a partial aortic clamping technique. Retrograde delivery of warm cardioplegia was not applied.

### 2.3. Endothelial Markers and Circulating Endothelial Cells

Assessment of markers of myocardial (CK-MB and troponin T) and endothelial (vWF, sTM, and CEC) injury was performed as previously described preoperatively and at 1 hour, 6 hours, 12 hours, and 24 hours after CPB initiation, respectively [14]. Briefly, blood was drawn from the central venous catheter and collected in citrate tubes for determination of vWF and sTM and centrifugated at 1000 g for 20 minutes at 4°C (Allegra X-15R, Beckman Coulter), and supernatant was frozen at –80°C. Concentrations of vWF (AssayMax Human von Willebrand Factor ELISA Kit, Assaypro, St. Charles, USA) and sTM (Human CD141 ELISA Kit, Diaclone SAS, Besançon, France) were assessed in triplicate measurements by a quantitative sandwich enzyme immunoassay technique, for which commercial ELISA kits were used.

For CEC isolation, blood was collected in EDTA tubes and measurements were immediately carried out using M-450 Dynabeads, 4.5 *μ*m diameter polystyrene beads coated with rat anti-mouse immunoglobulin-G1 (Dynal, Hamburg, Germany) coupled with murine anti-human CD146 antibody (Biocytex, Marseilles, France). Blood and phosphate-buffered saline containing 0.1% bovine serum albumin were mixed (1 mL each) with 20 *μ*L FcR blocking agent (Miltenyi, Bergisch-Gladbach, Germany) and incubated with 100 *μ*L anti-CD146-coated Dynabeads (8 × 10^6^ beads/10 *μ*L anti-CD146) for 60 minutes while agitating. Magnet separation (Dynal MPC) of cells bound to anti-CD146-coupled beads from blood was followed by washing with PBS-BSA-solution and incubation for 1 hour with 10 *μ*L of rhodamine-labeled Ulex-Europaeus-Agglutinin-1 solution (1 : 10 dilution, Linaris, Wertheim, Germany) on an orbital shaker in darkness. After washing and resuspension in 100 *μ*L of PBS-BSA, cells were counted in a Nageotte chamber (Brand, Wertheim, Germany) with a fluorescent microscope (Leica DMLB, Leica Microsystems GmbH, Wetzlar, Germany) using an excitation filter N2. CECs were identified and then counted as well-delineated round or oval rhodamine-labeled cells with the size of 10 to 40 *μ*m with more than 4 beads attached.

Samples for CK-MB and troponin T analysis were assessed at the Central Core Laboratory of the University of Cologne using commercially available assays for CK-MB (CK-NAC method, Roche Diagnostics, Mannheim, Germany) and cardiac troponin T determination (Elecsys troponin T, Roche Diagnostics). PMI was defined as the combination of (1) creatine kinase- (CK-) MB level 5 times greater than the upper level of normal (>120 U/L) with a CK-MB fraction between 6 and 25% and (2) a cardiac troponin T elevation greater than 1.5 ng/mL during the first 72 h after surgery [15, 16]. Major adverse cardiac events (MACE) were recorded as a composite endpoint including cardiac cause of death, PMI, inotropic support with epinephrine >24 hours (h), postoperative need for IABP or ECMO, and severe ventricular arrhythmias. The components of this endpoint were chosen to ensure high sensitivity for detection of complicated postoperative course.

### 2.4. Statistical Analysis

For statistical analysis, SPSS statistical software (SPSS Inc., Chicago, IL, US) was used. Categorical variables of groups were compared with Fisher's exact test and expressed as percentages. Continuous variables, given as mean ± standard deviation, were compared between groups by using the unpaired *t*-test for normally distributed values; otherwise, the Mann-Whitney *U* test was used. Variables with nonnormal distribution were presented as median and interquartile range (IQR). Two-way analysis of variance (ANOVA) with post hoc Bonferroni test was used for repeated measurements. *P* values <0.05 were considered to be statistically significant.

## 3. Results

### 3.1. Group Characteristics

Table 1 shows patients' demographic and operative data. Patients in the IWC-group (*n* = 36) presented with higher EuroSCORE and stayed longer on the ICU compared to patients treated with ICC (*n* = 32), but groups did not differ in terms of age, gender, preoperative EF, and NYHA-class. Similarly, procedure-related variables such as cross clamp time, total procedure time, and number of grafts implanted as well as postoperative ventilation times were comparable among groups, whereas CPB times were longer for ICC-patients. Postoperative incidence of atrial fibrillation was comparable for patients in the ICC- (23.1%) and IWC-group (28.6%; *P* = 0.772), respectively. Outcome variables PMI, MACE, and mortality were not detected in both treatment arms.

### 3.2. Markers of Myocardial Injury

**Figure 1:**
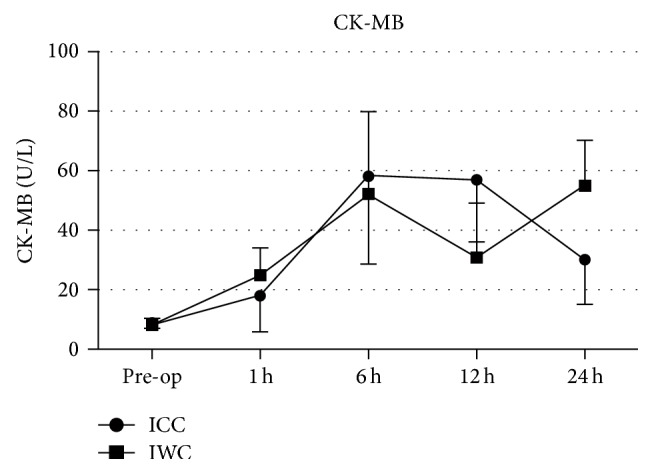
Course of values for CK-MB in the ICC-group and IWC-group preoperatively and at 1 h, 6 h, 12 h, and 24 h after the procedure.

The course of CK-MB values for patients in the ICC- and IWC-group is depicted in Figure 1. For both groups, CK-MB concentrations rose uniformly from normal preoperative values to peak concentrations at 6 hours after CPB initiation (ICC: 58.3 ± 29.7 U/L; IWC: 52.3 ± 27.5 U/L; *P* = 0.392). Although CK-MB concentration dropped after 12 hours in IWC-group, values were not significantly different.

**Figure 2:**
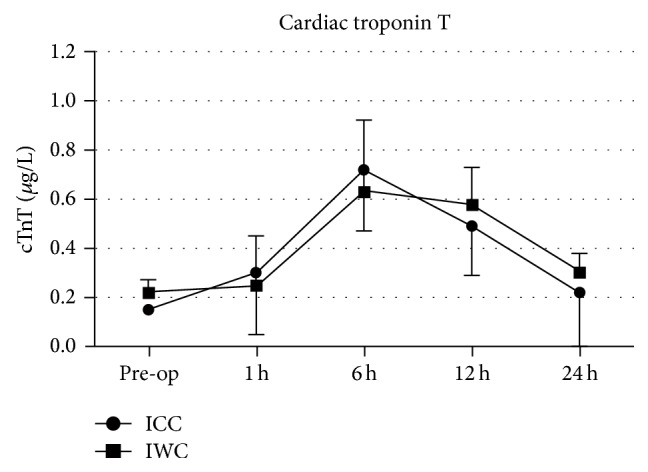
Course of values for cardiac troponin T (cTnT, *µ*g/L) in the ICC-group and IWC-group preoperatively and at 1 h, 6 h, 12 h, and 24 h after the procedure.

Cardiac troponin T levels for ICC- and IWC-groups are displayed in Figure 2. Parallel to CK-MB, cTnT concentrations of both groups peaked at 6 hours (ICC: 0.72 ± 0.25 *μ*g/L; IWC: 0.63 ± 0.29 *μ*g/L; *P* = 0.174) and declined uniformly in both groups without significant difference.

### 3.3. Markers of Endothelial Injury

**Figure 3:**
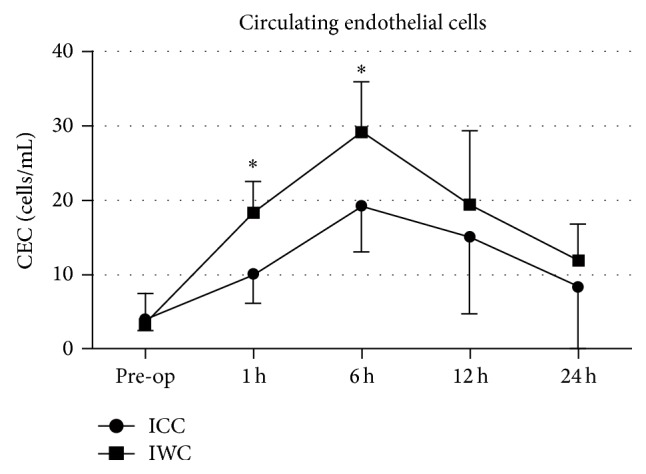
Number of circulating endothelial cells (CEC, cells per milliliter of blood) in the ICC-group and IWC-group preoperatively and at 1 h, 6 h, 12 h, and 24 h after the procedure.

Preoperative CEC counts (cells per milliliter of blood) were comparable among treatment groups (ICC: 3.9 (IQR: 2.8–4.6)/mL; IWC: 2.5 (IQR: 0.9–3.6)/mL; *P* = 0.488). However, CEC counts augmented in both groups and peaked at 6 hours after CPB initiation and nearly approached preoperative levels after 24 hours. Of note, CEC counts were significantly higher at 1 hour (ICC: 10.1 ± 3.9/mL; IWC: 18.4 ± 4.1/mL; *P* = 0.012) and 6 hours (ICC: 19.3 ± 6.2/mL; IWC: 29.2 ± 6.7/mL; *P* < 0.001) after CPB initiation in IWC-treated patients compared to ICC-group (Figure 3).

**Figure 4:**
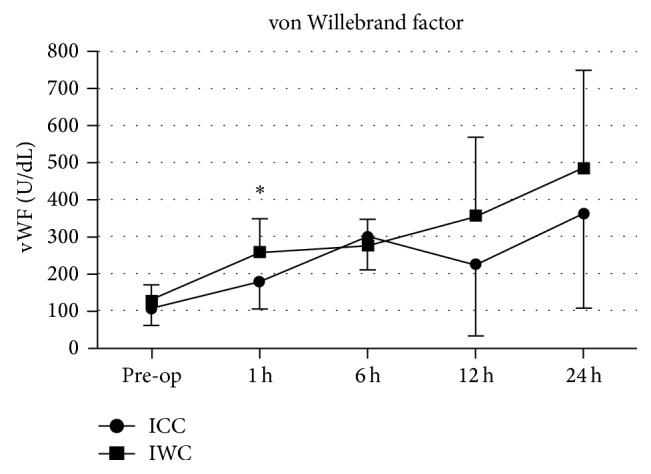
Concentration of von Willebrand factor (vWF, U/dL) in the ICC-group and IWC-group preoperatively and at 1 h, 6 h, 12 h, and 24 h after the procedure.

Measurements of vWF-concentrations showed rising trends uniformly for both groups with significant higher values at 1 hour after CPB initiation for IWC-group compared to the ICC-group (ICC: 178.4 ± 73.2 U/dL; IWC: 258.2 ± 89.7 U/dL; *P* < 0.001; Figure 4).

**Figure 5:**
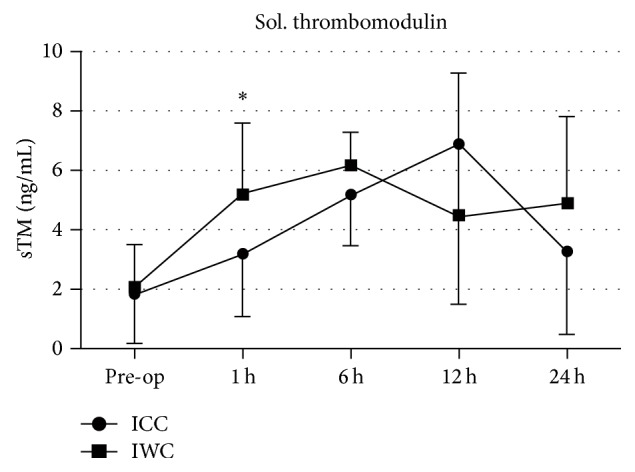
Concentration of soluble thrombomodulin (sTM, ng/mL) in the ICC-group and IWC-group preoperatively and at 1 h, 6 h, 12 h, and 24 h after the procedure.

Quantification of sTM concentration of patients in the ICC- and IWC-group revealed rising values with a peak at 6 hours after CPB initiation for IWC-patients and a peak at 12 hours for ICC-patients. Parallel to findings of vWF, values of patients in the IWC-group were significantly higher compared to ICC-patients at 1 hour after CPB initiation (ICC: 3.2 ± 2.1 ng/mL; IWC: 5.2 ± 2.4 ng/mL; *P* = 0.011; Figure 5).

## 4. Discussion

The present randomized and blinded single-center study assessed the impact of two routinely applied blood cardioplegic solutions on myocardial and endothelial injury in patients undergoing on-pump coronary artery bypass surgery. In this patient population with comparable myocardial damage as assessed by postoperative CK-MB and cardiac troponin T levels we demonstrate a slightly higher endothelial injury in patients treated with intermittent warm cardioplegia compared to intermittent cold cardioplegia. However, the detected differences do not allow preferring one solution over the other during elective CABG surgery in patients with average risk profile.

For determination of endothelial function in cardiac surgery patients, elevated numbers of circulating endothelial cells (CECs) and endothelium-specific plasma markers such as von Willebrand factor (vWF) and soluble thrombomodulin (sTM) have been shown to correlate with global vascular injury [9]. In patients undergoing on-pump cardiac procedures, Schmid et al. demonstrated the link of endothelial injury caused by cardiopulmonary bypass and blood levels of CECs since concentrations were significantly elevated 6 hours postoperatively compared to preoperative levels with a declining trend afterwards [17]. Similar to these findings, we generally detected rising concentrations of markers for endothelial injury with peak values of CECs at 6 hours postoperatively reflecting significant vascular damage triggered by the cardiac procedure. According to results of Skrabal et al. who assessed endothelial injury during on-pump CABG operations, the disturbance of the endothelium does not seem to be related to the mechanical manipulation of the heart itself, since levels of each of CECs, vWF, and sTM were unchanged 30 minutes after initiation of cardiopulmonary bypass but ascended in the postoperative period as a potential result of reperfusion injury [14]. This reperfusion injury comprises an activation of neutrophils, release of cytokines and proteases triggered by the contact of blood cells with artificial surfaces, and air during cardiopulmonary bypass finally interacting with the integrity of the endothelium [18].

Myocardial injury of included patients in the presented study did not differ between ICC- and IWC-group shown by similar levels of CK-MB and cardiac troponin T levels. Otherwise, endothelial injury was more pronounced in the IWC-group as we detected higher concentrations of markers for vascular damage compared to ICC-patients. Furthermore, significantly higher concentrations were uniformly detected of CECs, vWF, and sTM 1 hour after surgery for IWC-patients, while markers of myocardial damage were similar for both treatment groups. Therefore, the early rise of all markers for endothelial damage and the peak level of CECs 6 hours postoperatively might indicate a potential value of CECs as a super-sensitive marker for endothelial injury in cardiac surgical patients. In consequence, a lower endothelial damage in the ICC-group might indicate a superior myocardial protection when compared to IWC-use in this patient cohort undergoing CABG procedures, even though this can just be seen as a hypothesis-generating aspect. However, this finding would be in line with a study investigating the impact of ICC and IWC in high-risk patients subjected to a wide spectrum of different cardiac procedures with cross clamp times longer than 90 minutes. Use of IWC resulted in significantly higher cardiac mortality and in a more pronounced myocardial injury compared to ICC. Furthermore, application of IWC even turned out to be an independent predictor for 30-day all-cause mortality, cardiac death, and perioperative myocardial injury after multivariate analysis in this cohort [5]. It is obviously needed to underline the fact that the patient cohort in the presented study was different in terms of cross clamp time and risk profile. Otherwise, Franke et al. analyzed laboratory and clinical outcomes in a low-risk patient cohort undergoing exclusively CABG surgery. Markers of myocardial ischemia (CK-MB and cTnT) were significantly lower in patients treated with IWC when compared to ICC, whereas no differences in clinical endpoints were detected [4]. These contradicting results implicate the relevance of the patients' preoperative risk profile, the kind of underlying disease, and the influence of the cross clamp time since prolonged cross clamp time serves as an independent predictor for mortality in cardiac surgery patients [19].

Measuring endothelial injury during CABG procedures is naturally associated with uncontrollable covariates leading to limitations of our study and unanswered questions. Thus, we cannot explain the marked increase of CK-MB values in IWC-patients at 24 hours postoperatively. As we did not perform coronary angiography routinely in the postoperative period, we are unable to explain this phenomenon. Additionally, blood sample acquisition from the coronary sinus would have been desirable for determination of endothelial injury exclusively in the heart instead of using the central venous catheter. However, a sample collection via the coronary sinus would have been impossible after the end of the procedure for further measurements at 24 hours postoperatively.

Furthermore, it is important to underline that it is impossible to create two identical groups of patients even by randomization. Thus, both groups show differences in demographic and preoperative data that might have influenced the overall result. This fact might also be affected by the limited number of participating patients. Since this work presents a pilot trial, it is now possible to perform a sample size calculation for future studies on this topic.

Additionally, the presented investigation was not powered to detect significant differences in vascular damage associated with various myocardial protection techniques. This study reveals rather more the perioperative kinetics of markers for endothelia damage.

## 5. Conclusions

In summary, we demonstrate that perioperative endothelial injury can be assessed by measuring circulating endothelial cells, von Willebrand factor, and soluble thrombomodulin in patients undergoing elective, on-pump myocardial revascularization indicating a slight benefit of intermittent cold over warm blood cardioplegia in contrast to the primary hypothesis. However, this advantage is not reflected in differences in markers of myocardial injury or even clinical endpoints. The results of the presented pilot trial can serve as an argument for the safe use of intermittent cold blood cardioplegia in routine CABG surgery and as a basis for further investigations.

## Figures and Tables

**Table 1:** Patients' demographic and operative data.

Criteria	ICC-group	IWC-group	*P* value
Number (*n*)	32	36	
Age (years)	65.9 ± 11.3	63.9 ± 7.7	*0.392 *
Male gender (%)	75	81	*0.486 *
Preoperative EF (%)	62 ± 19	56 ± 23	*0.249 *
NYHA-class	2.1 ± 0.6	2.2 ± 0.6	*0.495 *
EuroSCORE	3.7 ± 2.3	4.2 ± 2.4	*0.385 *

CPB time (min)	76.2 ± 16.4	68.7 ± 12.5	*0.037 *
Cross clamp time (min)	39.7 ± 11.7	42.6 ± 14.6	*0.373 *
Procedure time (min)	187.7 ± 39.2	197.5 ± 42.7	*0.330 *
Number of grafts	3.1 ± 0.6	3.3 ± 0.6	*0.175 *

Ventilation time (hours)	17.4 ± 6.2	14.7 ± 8.5	*0.144 *
ICU stay (hours)	39.1 ± 17.6	52.3 ± 23.2	*0.011 *
Atrial fibrillation (%)	23.1	28.6	*0.772 *
PMI	0	0	*— *
MACE	0	0	*— *
Mortality	0	0	*— *

CPB: cardiopulmonary bypass; EF: ejection fraction; ICC: intermittent cold blood cardioplegia; ICU: intensive care unit; IWC: intermittent warm blood cardioplegia; MACE: major adverse cardiac event; NYHA: New York Heart Association; PMI: perioperative myocardial injury.
